# Grizzlies and gazelles: Duty factor is an effective measure for categorizing running style in English Premier League soccer players

**DOI:** 10.3389/fspor.2022.939676

**Published:** 2022-08-09

**Authors:** Brian Hanley, Catherine B. Tucker, Liam Gallagher, Parag Parelkar, Liam Thomas, Rubén Crespo, Rob J. Price

**Affiliations:** ^1^Carnegie School of Sport, Leeds Beckett University, Leeds, United Kingdom; ^2^Leeds United Football Club, Leeds, United Kingdom

**Keywords:** coaching, football, kinetics, leg stiffness, testing

## Abstract

English Premier League soccer players run at multiple speeds throughout a game. The aim of this study was to assess how well the duty factor, a dimensionless ratio based on temporal variables, described running styles in professional soccer players. A total of 25 players ran on an instrumented treadmill at 12, 16, and 20 km/h. Spatiotemporal and ground reaction force data were recorded for 30 s at each speed; video data (500 Hz) were collected to determine footstrike patterns. In addition to correlation analysis amongst the 25 players, two groups (both *N* = 9) of high and low duty factors were compared. The duty factor was negatively correlated with peak vertical force, center of mass (CM) vertical displacement, and leg stiffness (*k*_leg_) at all speeds (*r* ≥ −0.51, *p* ≤ 0.009). The low duty factor group had shorter contact times, longer flight times, higher peak vertical forces, greater CM vertical displacement, and higher *k*_leg_ (*p* < 0.01). Among the high DF group players, eight were rearfoot strikers at all speeds, compared with three in the low group. The duty factor is an effective measure for categorizing soccer players as being on a continuum from terrestrial (high duty factor) to aerial (low duty factor) running styles, which we metaphorically refer to as “grizzlies” and “gazelles,” respectively. Because the duty factor distinguishes running style, there are implications for the training regimens of grizzlies and gazelles in soccer, and exercises to improve performance should be developed based on the biomechanical advantages of each spontaneous running style.

## Introduction

Successful teams in the English Premier League (EPL) rely on multifactorial training programs that reflect the complex physiological demands of professional soccer (Kelly et al., 2020). The important technical skills crucial for individual players' and their team's performances are impacted by physical performance attributes (Bush et al., 2015), which involve running for long distances, including high-intensity bursts and occasional short sprints (Rutzler and Worville, 2021). Apart from walking, gait speeds in soccer have been classified as jogging at speeds ranging from 7.2 to 14.3 km/h, running from 14.4 to 19.7 km/h, high-speed running from 19.8 to 25.1 km/h, and sprinting at speeds >25.1 km/h (Bradley et al., 2009). The importance of running in soccer means it is a key aspect of performance that requires monitoring and analyzing, including from a biomechanical perspective.

An important factor in running mechanics is leg stiffness (*k*_leg_); increased stiffness in running is important in reusing the elastic energy stored during the early stance loading phase (Butler et al., 2003; Burns et al., 2021) and is thus associated with the better running economy (Barnes et al., 2014). Vertical stiffness of the whole body (*k*_vert_) is also used as a global measure of stiffness, usually increasing with running speed whereas leg stiffness is more consistent (Brazier et al., 2019). In this regard, two main running styles have been previously described: aerial and terrestrial (Lussiana et al., 2017). Aerial runners have high leg stiffness, short ground contact, and optimized technique through the release of stored energy, whereas terrestrial runners have low leg stiffness and longer contact times, and are economical by propelling themselves forward rather than upward (Gindre et al., 2016; Sandford et al., 2019). In explaining these contrasting concepts of a spring-like vertical bouncing gait vs. a grounded horizontal gait (Gindre et al., 2016) in our conversations with coaches and medical staff, we have metaphorically described players with these styles as “gazelles” and “grizzlies,” respectively. These metaphors are based on the visual imagery of how these animals run, rather than body size or shape, as well as the contrast in running styles of cursorial gazelles (e.g., running on the forefoot, long flight times) with non-cursorial grizzly bears, which have a higher duty factor (Biancardi and Minetti, 2012; Shine et al., 2015). Considering the distance covered using high-intensity running is lower in the last 15 min of EPL games compared with the first 15 min (Bradley et al., 2009), different running styles might have an impact on which players press the opposition, complete the whole game, or are more effective as substitutes.

Leg stiffness and vertical stiffness are difficult to measure without advanced biomechanical equipment such as force plates, although estimates are possible using calculations of body mass, forward velocity, leg length, flight time, and contact time (Morin et al., 2005). In simplifying the categorization of running styles even further, the duty factor is a dimensionless ratio that carries similar information as the stiffness models but does not rely on the assumptions of the models used (van Oeveren et al., 2021). The duty factor is calculated as the proportion of stride time (contact time plus swing time) when the foot is in contact with the ground (Hanley et al., 2022) and is thus a similar concept to the stance:swing ratio (Tao et al., 2019), where higher duty factors reflect a greater relative contribution of the contact phase (Lussiana et al., 2019). In recreational distance runners, Patoz et al. (2019) found that the duty factor could be used to rank runners as a measure of running form, but whether this also applies to professional soccer players, who undertake multifactorial training and greater ranges of running pace within a game, from jogging to sprinting, has not yet been investigated.

The identification of running styles in soccer players could be useful for coaches in prescribing training that deals with either strengths or weaknesses, and using objective measures might be important in individualizing training programs (Patoz et al., 2019). It is difficult to measure stiffness values, for example, without advanced biomechanical equipment, and using simpler methods to prescribe running style would be beneficial. Although there are subjective methods that could be used (Gindre et al., 2016; Zhang et al., 2021), these rely on extensive coach familiarity. Instead, the duty factor, which requires measurement of contact time and flight time only, could be a suitable proxy for global biomechanical measures and hence the description of running styles. The aim of this study was to assess how well the duty factor describes running styles (including global measures of stiffness) during jogging (12 km/h), running (16 km/h), and high-speed running (20 km/h) in EPL soccer players.

## Materials and methods

### Research approval

The study was reviewed and approved by the Carnegie School of Sports Research Ethics Committee. All human subjects were over the age of 18 years and provided their written informed consent to participate. The study was conducted in accordance with the recognized ethical standards of the Declaration of Helsinki. In accordance with the Carnegie School of Sports Research Ethics Committee's policies for use of human subjects in research, all subjects were informed of the benefits and possible risks associated with participation and informed of their right to withdraw.

### Participants

A total of 25 male professional soccer players (age: 23.7 ± 4.1 years, height: 1.80 ± 0.07 m, mass: 75.7 ± 7 kg; leg length: 0.92 ± 0.04 m) from a single EPL club were tested during pre-season. Leg length was measured as the standing height of the greater trochanter. All participants have played in the EPL and were healthy and free from injury at the time of testing. A total of 12 players have played full senior internationals for their nation. All players were outfield players (i.e., goalkeepers were not included).

### Data collection

After a 6-min warm-up and familiarization period, each subject ran on a gaitway-3D instrumented treadmill (h/p/Cosmos, Traunstein, Germany) for an incremental test at 12, 16, and 20 km/h for 3 min each. To avoid fatigue, the players walked at 5 km/h for 3 min between stages, and the 3-min running stage included the time taken to gradually reach the target speed and slow down; for example, this meant that at the fastest speed of 20 km/h, the players ran at this speed for ~60 s. The treadmill's inclination was set at 0% during data collection (Paquette et al., 2017). The treadmill incorporated four in-dwelling three-dimensional strain gauge sensors (Arsalis, Louvain-la-Neuve, Belgium) that recorded GRF data (1,000 Hz) from both feet as well as temporal data. The treadmill also recorded the position of the center of pressure from which step length was measured. Data were collected for 30 s during each stage, which allowed for the collection of between 38 and 50 steps per foot during each sampling period, beginning 2 min after the commencement of the stage. The subjects wore no upper body covering, shorts, and running shoes and were accustomed to treadmill running. A large fan was placed in front of the subjects to help cool them.

To record footstrike patterns, two-dimensional video data were collected using a high-speed camera (Fastec, San Diego, CA) with a 25-mm fixed lens at 500 Hz (shutter speed: 1/1,000 s; *f*-ratio: 2.0; ISO: 3,200; FHD: 1,280 × 1,024 px). The camera was placed 3.15 m from and perpendicular to the treadmill. Extra illumination was provided by eight 750-W lights. The video recordings were synchronized with the start of kinetic data collection *via* a rising edge trigger.

### Data analysis

The GRF data were analyzed using the associated software (h/p/Cosmos, Traunstein, Germany) and smoothed using an eighth-order, low-pass Bessel filter. The optimal cut-off frequency was calculated during pilot testing using residual analysis (Winter, 2005); the results showed an optimal cut-off frequency ranging from 27.8 to 32.2 Hz, and so it was decided to use 30 Hz as the cut-off frequency for all trials. The collected video files were analyzed in SIMI Motion (version 9.2.2, Simi Reality Motion Systems GmbH, Germany). Footstrike patterns (first 10 steps) were defined using the foot position at initial contact using the methods of Hasegawa et al. (2007) as either rearfoot striking (the heel contacted the treadmill surface first without simultaneous contact by the midfoot or forefoot), midfoot striking (the heel and midfoot contacted simultaneously) or forefoot striking (the forefoot contacted first with a clear absence of heel contact).

Step length was the distance from one-foot strike at initial contact to the next foot strike of the opposite foot, and step frequency was the number of steps taken per second. Contact time was the time duration from initial contact to toe-off, and flight time was the time between contact phases during which no GRFs were recorded (the vertical force magnitude was less than the mean plus two standard deviations (SD) of the noise). Stride time was the duration between successive initial contacts of the same foot, and duty factor was the ratio of contact duration to stride duration. Vertical and leg stiffnesses were calculated using the equations of McMahon and Cheng (1990): vertical stiffness (*k*_vert_) was the ratio of the peak vGRF to CM vertical displacement; leg stiffness (*k*_leg_) was the ratio of the peak vGRF to the compression of the leg. CM vertical displacement was calculated as the maximum vertical displacement of the CM during a step and obtained by double integration of the vGRF signal. To account for differences in body size, vGRF data were normalized as bodyweights (BW) by dividing the magnitudes by the players' weights; *k*_vert_ and *k*_leg_ were normalized using leg length and body weight (McMahon and Cheng, 1990). All kinetic and spatiotemporal variables were averaged across left and right legs.

The duty factor for each participant was averaged across all running speeds to separate players into high and low duty factor groups (Lussiana et al., 2019). To establish these, the players were ranked for duty factor at each of the three running speeds, as well as the mean value. Those players who were always ranked in the top half for duty factor were considered the “high DF group”; those players who were always ranked in the bottom half were the “low DF group.” A total of seven players switched from a ranking in the top half to the bottom half across speeds or vice versa, and these players were excluded from both groups; they were also the middle-ranked players for mean duty factor (i.e., ranked 10–16th of the 25 players). Both high and low DF groups thus comprised nine players.

### Statistics

Statistical analyses were undertaken using SPSS Statistics 27 (IBM SPSS, Inc., Chicago, IL). The data were found to be normally distributed using a Shapiro-Wilk test. Between-group and within-group differences were determined using a two-way mixed-design ANOVA with the two DF groups and three speeds as the main factors, with Greenhouse-Geisser correction, used when Mauchly's test for sphericity was significant. Repeated contrast tests were used to establish significant differences between successive measurement speeds (Field, 2009); Cohen's *d* (Cohen, 1988) was used as an effect size to determine the magnitude of the differences between successive speeds and were either trivial (*d* < 0.2), small (0.21–0.6), moderate (0.61–1.2), large (1.21–2), very large (2.01–4) or extremely large (>4) (Hopkins et al., 2009). Pearson's product-moment correlation coefficient (*r*) was used to find associations between variables and duty factors, and were small (*r* = 0.1–0.29), moderate (0.3–0.49), large (0.5–0.69), very large (0.7–0.89), or extremely large (≥0.9) (Hopkins et al., 2009). An alpha level of 5% was set for all tests.

## Results

**Figure 1:**
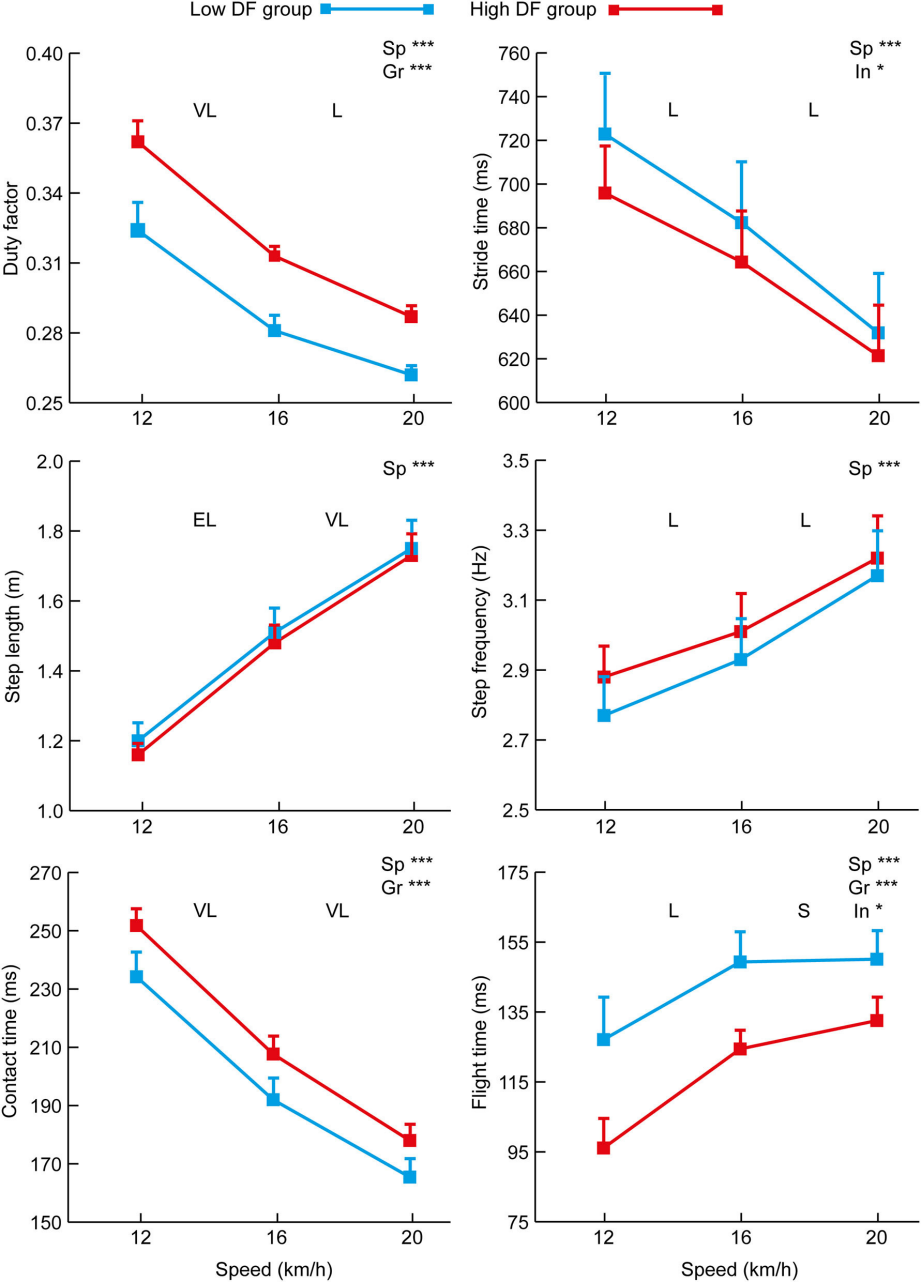
Mean (+SD) values for spatiotemporal data at each of the three testing speeds for the high and low DF groups. Overall effects for speed (“Sp”), group (“Gr”) and interaction (“In”) are annotated on each figure where statistically significant (*p* < 0.05 *, *p* < 0.01 **, or *p* < 0.001 ***). The effect size (Cohen's *d*) for differences between successive test speeds is annotated as small (S), moderate (M), large (L), very large (VL), or extremely large (EL).

**Figure 2:**
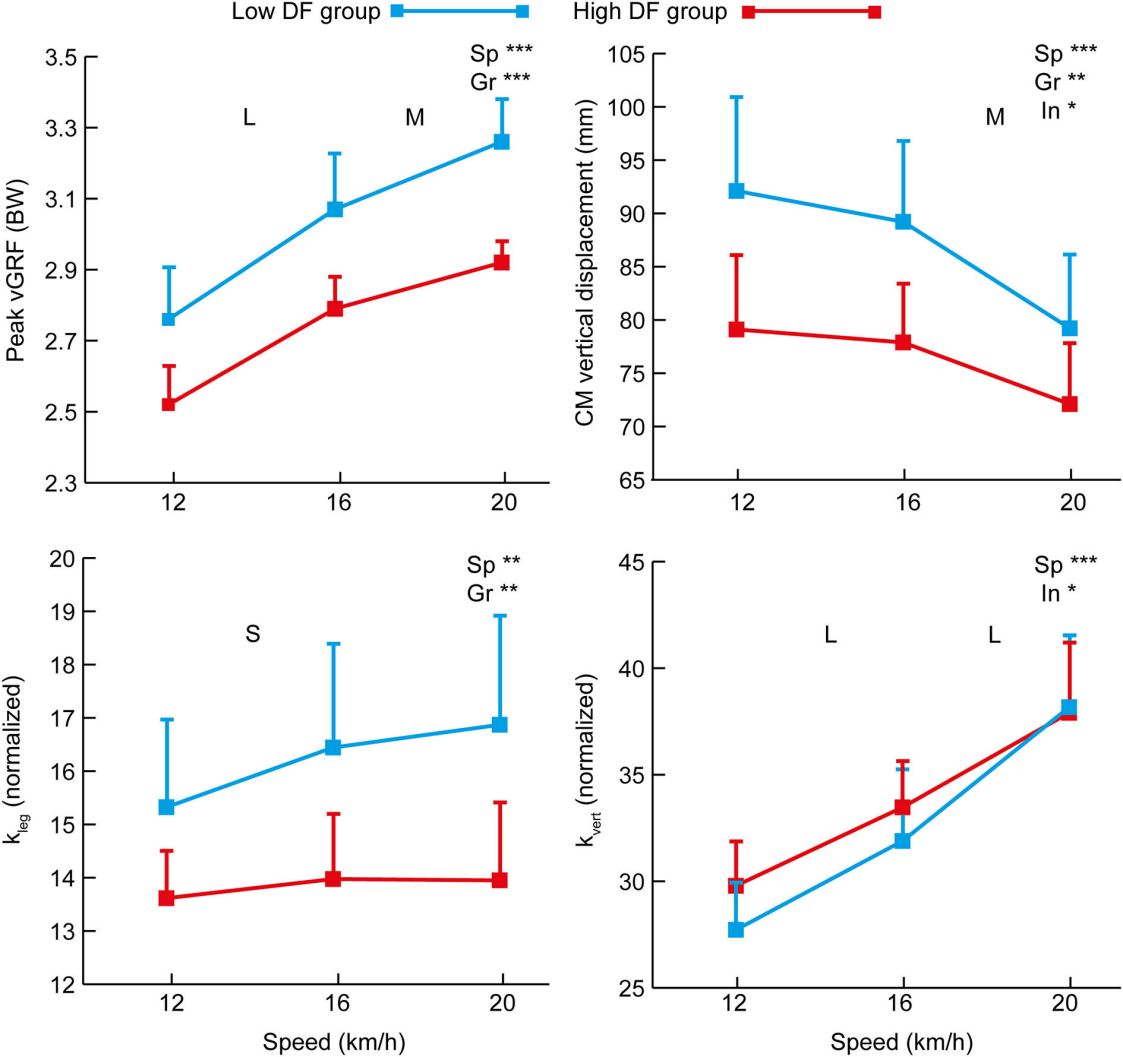
Mean (+SD) values for vGRF, CM vertical displacement, and global stiffness variables at each of the three testing speeds for the high and low DF groups. Overall effects for speed (“Sp”), group (“Gr”) and interaction (“In”) are annotated on each figure where statistically significant (*p* < 0.05 *, *p* < 0.01 **, or *p* < 0.001 ***). The effect size (Cohen's *d*) for differences between successive test speeds is annotated as small (S), moderate (M), large (L), very large (VL), or extremely large (EL).

The analysis of the two DF groups across speeds showed an overall effect of speed for duty factor, stride time, step length, step frequency, contact time, and flight time (Figure 1) as well as peak vGRF, CM vertical displacement, *k*_leg_ and *k*_vert_ (Figure 2) (*p* ≤ 0.001). There were differences between the DF groups for stride time, contact time, flight time, peak vertical force, CM vertical displacement, and *k*_leg_, and a speed x group interaction for stride time, flight time, CM vertical displacement, and *k*_vert_. The duty factor was correlated with *k*_vert_ at 12 km/h only (Table 1). The associations between flight time and duty factor were larger than for contact time. There were large or very large negative correlations between peak vertical force, CM vertical displacement, and *k*_leg_ with duty factor (Table 1).

**Table 1:** Significant correlations (*p* < 0.05) between duty factor and key variables for all 25 players at each of the three treadmill running speeds.

	**12 km/h**	**16 km/h**	**20 km/h**
Step length	*r* = −0.65	*r* = −0.40	
	*p* < 0.001	*p* = 0.047	
Step frequency	*r* = 0.63	*r* = 0.42	
	*p* < 0.001	*p* = 0.037	
Contact time	*r* = 0.77	*r* = 0.72	*r* = 0.59
	*p* < 0.001	*p* < 0.001	*p* = 0.002
Flight time	*r* = −0.98	*r* = −0.93	*r* = −0.87
	*p* < 0.001	*p* < 0.001	*p* < 0.001
Peak vGRF	*r* = −0.83	*r* = −0.83	*r* = −0.85
	*p* < 0.001	*p* < 0.001	*p* < 0.001
CM vertical displacement	*r* = −0.82	*r* = −0.71	*r* = −0.60
	*p* < 0.001	*p* < 0.001	*p* = 0.001
*k*_leg_	*r* = −0.51	*r* = −0.62	*r* = −0.59
	*p* = 0.009	*p* < 0.001	*p* = 0.002
*k*_vert_	*r* = 0.57		
	*p* = 0.003		

There was no difference in height, mass, or leg length between the high and low DF groups. The mean duty factor across the three speeds for the high DF group was 0.320 (±0.004) whereas for the low DF group it was 0.289 (±0.004). There was an overall effect of speed and group for the duty factor but no interaction (Figure 1). At 12, 16, and 20 km/h, the range of duty factors for the high DF group were 0.353–0.375, 0.309–0.318, and 0.280–0.294, respectively; for the low DF group, the ranges were 0.299–0.345, 0.269–0.294, and 0.257–0.270, respectively. Among the nine high DF group players, eight were rearfoot striking at all three speeds, with the other player rearfoot striking at 12 km/h but midfoot striking at the two faster speeds. Of the low DF group, three were rearfoot striking across all testing speeds, four were midfoot striking at all speeds, one was rearfoot striking at 12 and 16 km/h but midfoot striking at 20 km/h, and one was rearfoot striking at 12 km/h but midfoot striking at 16 and 20 km/h.

## Discussion

The aim of this study was to assess how well the duty factor describes running styles during jogging, running, and high-speed running in EPL soccer players. Overall, the results from the correlation analysis and the comparisons between high and low DF groups showed that the duty factor is a suitable measure to determine the running style. This supports the findings of Patoz et al. (2019) who categorized recreational distance runners but provides more support through the inclusion of global stiffness and kinetic measures rather than a visual analysis method. Duty factor correlated with *k*_leg_ at all three running speeds, as well as peak vGRF and CM vertical displacement (Lussiana et al., 2017). Duty factor can therefore be used as a descriptor of a soccer player's running style and indicate their preference for a terrestrial (“grizzly”) or aerial (“gazelle”) gait. The separation of the top- and bottom-ranked players for duty factor into high and low DF groups emphasized their different running mechanics, especially regarding contact and flight times, peak vGRF, CM vertical displacement, and *k*_leg_. Coaches who wish to incorporate running style to inform individualized training programs can use the duty factor to categorize them as having either a “grizzly” or “gazelle” running style within that team (or somewhere in between). We found no differences in stature, mass, or leg length between high and low DF groups, and so these category names are intended as visual metaphors of running styles rather than body size.

It was found that most players were consistently either terrestrial or aerial runners. This concurs with previous research where *k*_leg_ was relatively constant (Heise and Martin, 1998; Hobara et al., 2010), and with the results of this study that showed only a small increase in *k*_leg_ across running speeds despite decreases in duty factor. However, there was a considerable range in the increase for *k*_leg_ within this group, with one player's values increasing by 30% between 12 and 20 km/h, and whose duty factor also decreased the most of all players (by 26%) between these speeds. This player, therefore, re-uses energy better at faster speeds than other players in the sample, similar to how a kangaroo possesses elastic structures that return more energy at higher than at lower speeds (Lussiana et al., 2019). Given that aerial runners might be relatively more economical than terrestrial runners at faster speeds, and terrestrial runners relatively more economical than aerial runners at slower speeds (Lussiana et al., 2017), it is conceivable that aerial players are able to optimize running costs across speeds better by adopting the grizzly style when running slowly, and the gazelle style when required to run faster. Within their multifactorial training programs, it seems sensible for soccer players to improve aspects of both terrestrial and aerial running styles to manage better the demands of different running speeds, and to focus not only on their strengths but also on their weaknesses. However, this does not imply that the exercises and sessions used to develop particular physical attributes should be identical for players with either aerial or terrestrial preferences. For instance, if the coach wishes to improve the rebound qualities of each player, the methods used should differ so that the advantages of their spontaneous aerial or terrestrial preference are exploited.

In terms of practical applications, it has been suggested that there is a generally beneficial set of mechanical parameters for each running style that develop the underlying joint stiffness (Brazier et al., 2019). It has therefore been suggested that plyometric training, which enhances *k*_vert_ and *k*_leg_ (Brazier et al., 2019), might suit the aerial patterns of “gazelles” better, whereas resistance training (e.g., lifting weights) might be more relevant for running style of the terrestrial “grizzlies” (Gindre et al., 2016), especially as the latter running style minimizes flight time to make it easier to change direction quickly (Struzik et al., 2021). Terrestrial soccer players' greater reliance on the contact phase and their shorter flight times suggest that they rely on more forward impulse per step (Taylor and Beneke, 2012; Sandford et al., 2019), which could therefore be better suited to the repeated, short sprints that are characteristic of soccer. Indeed, “Groucho running,” where the knee is more flexed than usual (McMahon et al., 1987), is often adopted in team sports to facilitate changes in direction, but the reduced *k*_vert_ and *k*_leg_ that ensue increase oxygen consumption (Struzik et al., 2021). Lower duty factors have been found to be associated with the better running economy (Folland et al., 2017) and, in-game situations, the “gazelle”-style players might be therefore more suited to continuous box-to-box running, whereas the “grizzlies” would appear more suited to short sprints and marking opponents because their relatively lower lift of the CM allows for easier changes in direction (Struzik et al., 2021). It is not being suggested that either type of player is better, but rather that they have different strengths and weaknesses and modalities for optimizing performance; for example, energy cost is minimized by the aerial “gazelle” players by optimizing the spring-mass model, and by the terrestrial “grizzly” players by limiting CM vertical displacement to prioritize forward movement (Lussiana et al., 2019). Ultimately, either type of player benefits from training modalities that develop passive energy usage or improve muscle force-generating capacity, and which could be specific to different lower limb joints (e.g., ankle, hip) (Struzik et al., 2021), but which exact exercise is best could be influenced by their spontaneous running style.

It was unsurprising that increased running speed meant changes in key deterministic variables including step length, step frequency, contact time, and flight time. It is clear that deciding which duty factor value to use in categorizing a player's running style cannot be based on a single speed. The running speeds chosen for this study were within the ranges described for jogging, running, and high-speed running (Bradley et al., 2009), but other speeds could be used that reflect sprinting speeds (>25.1 km/h) (Bradley et al., 2009) that were not possible using the treadmill protocol, and the typical paces of specific players analyzed (e.g., women, youth players). Indeed, the particular team analyzed was noteworthy during the previous EPL season for long-distance running at higher speeds (Rutzler and Worville, 2021) and might be much more able for this quantity of running than even other professional teams; thus, alternative methods of measuring duty factor (e.g., on grass) could be more suitable. One noticeable aspect was that more gait variables were correlated with duty factor at 12 km/h than at 16 or 20 km/h and, without the results from the faster speeds, these associations could be misleading in terms of what factors can indicate running style. For instance, step length and step frequency were not associated with duty factor at 20 km/h, and given these variables did not differ between high and low DF groups, could be less useful indicators of running style. Although 72% of players who were in either the top or bottom 50% for duty factor at the slowest speed were also in that half for the faster speeds, approximately a quarter of the players switched between the bottom and top halves, or vice versa, and using a mean value of three distinct running speeds was a better guide to overall running style (grizzly, gazelle or kangaroo). Duty factors can be measured using field-based equipment such as OptoJump (Brazier et al., 2019). However, practitioners should note the need to adjust the sensitivity settings of this system to accurately measure temporal variables when running overground or on a treadmill (Hanley and Tucker, 2019). Similarly, app-based measurements could be made (Romero-Franco et al., 2017), but regardless of what system is used, a sufficient number of steps at a relatively constant speed are needed for analysis.

Soccer is predominantly played on a yielding grass surface, and so a limitation of this study is that treadmill running limits the external validity of the results as the players effectively ran in a straight line, the surface was consistently firm, and the footwear used was not the boots worn in games or outdoor training. However, using a treadmill was important for internal validity by obtaining data at the specified running speeds and maintaining environmental constraints so that speed was the only independent variable altered. The instrumented treadmill allowed for a high number of steps to be recorded per trial and meant that robust kinetic and spatiotemporal data could be collected. There are potential differences between overground and treadmill gait (van Hooren et al., 2020) regardless of differences in footwear and surface stability, and field-based testing of running style (e.g., on grass) will likely provide different results for duty factor. Future studies that assess the similarity between overground and treadmill running in assessing gait biomechanics in soccer players would therefore be useful in understanding the impact of different shoe-surface interactions on running styles, and field-based studies on players' gait in football boots would provide ecologically valid measures of duty factor and help demonstrate its value in describing running styles in soccer, including at sprinting speeds.

## Conclusions

This novel study found that measuring duty factor during running is a simple but effective measure for categorizing professional soccer players on a continuum from terrestrial (“grizzly”) to aerial (“gazelle”) running styles, although it should be noted that 28% of the players could not be consistently categorized in either group and shows that there is a range of duty factors on which a player can lie. Because of their preference for using the contact phase for developing forward propulsion, rather than upward, the terrestrial players had common characteristics like higher duty factor, lower *k*_leg_, lower peak vGRF, and less CM vertical displacement than their aerial counterparts. Although soccer training is typically multifactorial in dealing with strengths and weaknesses, players found to prefer the terrestrial style of running might be more suited to aspects of play related to changing direction and great acceleration, whereas those with an aerial style have a style more similar to long-distance runners and could therefore find it physically easier to cover greater distances in games and training. Coaches should consider adopting different exercises for players depending on spontaneous running style even when trying to achieve the same physical improvement within the team.

## Data availability statement

The raw data supporting the conclusions of this article will be made available by the authors, without undue reservation.

## Ethics statement

The studies involving human participants were reviewed and approved by Carnegie School of Sport Research Ethics Committee. The patients/participants provided their written informed consent to participate in this study.

## Author contributions

RC and RP arranged data collection within the football club. BH, CT, PP, and LG performed data collection. BH and CT processed the data and BH created the figures. All authors conceptualized and designed the study, wrote the manuscript, interpreted the results of the research, edited, critically revised, and approved the final version for submission.

## Funding

The data collection was supported by funding provided by Leeds United Football Club as part of a wider sports science support project; however, the nature of the data is purely descriptive and not associated with any governing body, commercial sector, or product. No funding was provided for the writing of this article. The results of this study do not constitute an endorsement by Leeds United Football Club. The funder was not involved in the study design, collection, analysis, interpretation of data, the writing of this article or the decision to submit it for publication.

## Conflict of interest

Authors RC and RP were employed by Leeds United Football Club. The authors declare that the research was conducted in the absence of any commercial or financial relationships that could be construed as a potential conflict of interest.

## Publisher's note

All claims expressed in this article are solely those of the authors and do not necessarily represent those of their affiliated organizations, or those of the publisher, the editors and the reviewers. Any product that may be evaluated in this article, or claim that may be made by its manufacturer, is not guaranteed or endorsed by the publisher.
